# Embedded implementation research determinants in Latin American health systems

**DOI:** 10.11606/s1518-8787.2021055003027

**Published:** 2021-04-08

**Authors:** Rodrigo García-Cerde, Victor Becerril-Montekio, Étienne Langlois, Ludovic Reveiz, Jacqueline Alcalde-Rabanal, Pilar Torres-Pereda

**Affiliations:** I Instituto Nacional de Salud Pública Centro de Investigación en Sistemas de Salud CuernavacaMorelos México Centro de Investigación en Sistemas de Salud. Instituto Nacional de Salud Pública. Cuernavaca, Morelos, México.; II World Health Organization Geneva Switzerland Partnership for Maternal Newborn and Child Health. World Health Organization. Geneva, Switzerland.; III Pan American Health Organization Department of Evidence and Intelligence for Action in Health WashingtonD.C. USA Department of Evidence and Intelligence for Action in Health, Pan American Health Organization, Washington, D.C, USA.

**Keywords:** Public Health Systems Research, Research Support as Topic, South-South Cooperation, Qualitative Research

## Abstract

**OBJECTIVE:**

To assess the determinants of embedded implementation research (EIR) conduct in seven Latin American and Caribbean countries.

**METHODS:**

This qualitative interpretative study conducted and analyzed 14 semi-structured interviews based on a grounded theory approach using Atlas-ti© 7.5.7. We grouped the conditions appointed by interviewees as determinants of EIR conduct into six domains.

**RESULTS:**

The participation of high-level engaged decision makers as research co-producers is an important EIR determinant that fosters research use. Nevertheless, EIR faces challenges such as dealing with key personnel changes and fluctuating political contexts.

**CONCLUSIONS:**

Despite its limitations, EIR is effective in creating a sense of ownership of research results among implementers, which helps bridge the gap between research and decision-making in health systems.

## INTRODUCTION

Most efforts to put scientific evidence in the hands of policymakers and health system managers to ensure evidence-based informed decision making have been based on one-way communication from researchers to decision makers^1^. Generally described as knowledge translation^2^^,^^3^, its general objective is to present research results using language and forms accessible to decision makers who, eventually, will use it.

But empirical findings from studies have not always answered policy-relevant questions, mostly because end-users were not involved in the research cycle from the beginning. To meet these challenges, new innovative forms of collaboration between researchers and decision makers have been developed. Initially, they were limited to consulting decision makers in search for a common research agenda and setting priorities^4^.

More recent approaches try to include research results users as active partners throughout the research process, under different names: collaborative research, engaged research, participatory research, research co-production, integrated knowledge translation, or, as in our study, embedded research (ER)^1^^,^^5^^–^^7^.

Embedded research can also be understood as integrating research itself within organizations to ensure that the entire research process is conducted collaboratively by the health personnel along with professional researchers, creating a synergy based on their mutual expertise to improve implementation. Research and policy social actors thus participate together in identifying and defining research problems, as well as designing and conducting the research, analyzing, disseminating and accepting results^8^.

The main objective of implementation research (IR), which is particularly linked to embedded research, is to improve program implementation and not just to advance knowledge production^9^. According to this perspective, decision makers and implementers have relevant knowledge gained by their daily experience that is directly useful for improving implementation. As shown in several studies, IR is well-suited for embedding research in health programs, policies and services^10^.

On these bases, in 2014 and 2016 the Pan American Health Organization (PAHO) and the Alliance for Health Policy and Systems Research (AHPSR) launched the Improving Program Implementation through Embedded Research (iPIER) initiative to strengthen program implementation and provide support to research projects conducted by decision-makers and researchers in Latin American and Caribbean (LAC) countries^11^^,^^12^. It funded implementation research projects, mostly in public health institutions, where a decision maker led a large team including other health staff and local researchers. The initiative focused on capacity strengthening at the individual and institutional levels, based on workshops and ongoing technical support provided by researchers from the National Institute of Public Health of Mexico, co-authors of this paper.

Embedding research in health programs, policies and services is a complex process that requires deeper understanding. Certain conditions may favor or hinder its implementation and have yet to be sufficiently examined, needing further discussion and new perspectives. Existing knowledge gaps have to be bridged to make the most of this kind of efforts^13^. These conditions refer to some of the domains of the Consolidated Framework for Implementation Research – CFIR^14^, mainly the outer and inner settings and the characteristics of the participating individuals.

This article draws on the iPIER 2016 experience with seven decision-maker led teams in Argentina, Bolivia, Brazil, Chile, Colombia, Peru and Dominican Republic. Each team focused their research on existing health programs, policies and services searching for the best ways to improve their implementation (Table 1). Our aim is to analyze the different conditions that influence, facilitate or hinder the development of embedded implementation research in Latin American and Caribbean countries.

**Table 1:** Main findings and recommendations of the projects developed by IPIER participants.

Country (C) *	Project theme	Level	Main implementation problems identified	Main recommendations
C1	Promotion of male-centered family planning	National	Lack of specific actions focused on men. Lack of training of health personnel to offer sexual education and contraceptive methods to young men. The spaces are unattractive to men, as they are installed in maternity services.	Promote adjustment in the normative guidelines. Train staff to address men. Adaptation of spaces in health services for the adequate care of men's sexual and reproductive health.
C2	Tuberculosis care in marginal urban areas	Regional	Lack of operational guidelines. Lack of community organizations’ involvement. Staff have stigmatizing perceptions of working with people living with tuberculosis.	Develop manuals and operational guidelines. Give a more active role to the civil society. Develop strategies to reduce stigma among health staff.
C3	Cervical cancer screening and care	Local	Lack of prioritization of cervical cancer. Ignorance of the program and users’ unfavorable beliefs on its use. Lack of monitoring and evaluation of the program.	Name a program responsible at the municipal level. Produce informative material. Staff training on adequate and warm counseling. Establish monitoring and evaluation mechanisms.
C4	Normative regulation of medicines’ bio-equivalence	National	Lack of coordination among actors during the policy design. Insufficient monitoring mechanisms. Increase of generic drug prices as a result of the policy.	Participatory discussion process with key actors to implement the policy. Train and qualify the Public Health Institute on monitoring and supervision activities. Evaluate the possible policy effects.
C5	Promotion of a psycho-social mental healthcare model	Local	Lack of procedure registration. Lack of users’ prominence in psychosocial healthcare units. Ignorance of users’ rights.	Train staff on proper management. Promote civic and institutional activities to raise awareness on the users’ role in their own healthcare.
C6	Malnutrition care in infants	Regional	Socio-cultural problems: lack of mothers’ time, dislike of the micronutrients’ taste, lack of understanding on the micronutrients importance. Institutional problems: shortage, lack of time to provide guidance.	Identify socio-culturally appropriate alternatives for the intake and consumption of micronutrients in infants. Collaboration with other programs.
C7	Tuberculosis healthcare in prisons	Regional	Lack of access to health services with a right to health perspective. Lack of coordination between the provincial health ministry and the provincial justice ministry.	Provide training on tuberculosis and human rights and design a protocol of actions to detect damage to the inmates’ health. Propose an inter-ministerial collaboration agreement.

* We replaced the names of countries to assure the anonymity of informants.

## METHODS

### Design

Ours is a qualitative-interpretative study based on the methodological postulates of grounded theory^15^, using thematic analysis. The semi-structured interviews explored the perceptions of key informants on the conditions that facilitated or hindered EIR in their contexts, and the possibilities of using the results derived from research projects.

We conducted two interviews per team, one during the first stages of the research projects and a second at the end. As our aim was not to evaluate changes derived from the IPIER initiative itself, we do not differentiate them as baseline and follow-up.

An anthropologist with a master's degree in Public Health and Epidemiology and no ties to the participating institutions conducted both the interviews and qualitative analysis. Before each interview, the researcher introduced himself and explained the purpose of the interviews and the study. The authors had no previous connections with the study participants.

### Participants and Study Setting

Participants’ selection criteria included being the research project leader or co-investigator and voluntarily accepting to participate in the study based on an informed consent procedure. We assured privacy for all semi-structured interviews held, interviewing one or more people from each team according to their interest. In the end, 15 informants participated in the interviews and no one declined (Table 2).

**Table 2:** Characteristics of study participants (n = 15).*

Participant	Gender (F / M)	Country identifier	Interview (1^st^ / 2^nd^)	Type of Institution	Role in the Research Team (PI /CI)	Bachelor education
A	Female	C7	1^st^	Public Management	Principal Investigator / Decision Maker	Medicine
B	Female	C7	2^nd^	Independent Consultant	Co – Investigator / Researcher	Political Sciences
C	Female	C6	1^st^ & 2^nd^	Public Health Services	Principal Investigator / Researcher	Medicine
D	Male	C5	1^st^ & 2^nd^	Academic Institution	Principal Investigator / Decision Maker	Psychology
E	Female	C5	1^st^ & 2^nd^	Academic Institution	Co-investigator / Researcher	Psychology
F	Male	C4	1^st^	Public Management	Co-investigator / Researcher	Industrial engineering
G	Male	C4	2^nd^	Independent Consultant	Co-investigator / Researcher	Anthropology
H	Male	C3	1^st^	Public Health Services	Co-investigator / Researcher	Medicine
I	Female	C3	2^nd^	Public Health Services	Principal Investigator / Decision Maker	Bacteriology
J	Female	C2	1^st^ & 2^nd^	Academic Institution	Principal Investigator / Decision Maker	Psychology
K	Female	C2	1^st^ & 2^nd^	Academic Institution	Co-investigator / Researcher	Medicine
L	Female	C1	1^st^ & 2^nd^	Public Management	Principal Investigator / Decision Maker	Medicine
M	Female	C1	1^st^	Academic Institution	Co-investigator / Researcher	Medicine
N	Female	C1	2^nd^	Academic Institution	Co-investigator / Researcher	Sociology
O	Female	C1	2^nd^	Public Management	Co-investigator / Researcher	Medicine

* Only the characteristics of the people who participated in the interviews are presented, but each team consisted of more collaborators. The name of each participant's country was hidden to protect their anonymity. Identifications will be presented as follows: PA = participant A, F/M = Female, Male, C1 = Country 1, PI/CI = Principal Investigator, Co-Investigator, Bachelor education.

### Data Collection

Interviews took place between October 2016 and November 2017, during and after research activities. We conducted a total of 14 semi-structured interviews in Spanishᵃ, two for each team, following two interview guides (Appendix 1). The first interview took place while the participants were finishing their protocol or starting fieldwork; the second, when they were writing their final report. According to what each team considered appropriate, the same or different people participated in the first and second interviews. During on average one hour, all interviews were audio recorded and transcribed with the informants’ agreement. At the end of each interview, the researcher wrote analysis notes.

### Data Analysis

We performed a thematic analysis^16^ based on grounded theory principles to process the narratives with axial coding^15^, using Atlas-ti^©^ 7.5.7 software. After interviewing the participants and codifying the transcriptions, both done by the same researcher, we performed an interpretative triangulation with other researchers reviewing the testimonies.

We decided to use an a priori “self-selected” sample to draw lessons to improve the development of initiatives such as iPIER and to provide further evidence on EIR usefulness. We thus observe that the identified theoretical saturation and explanatory density^17^ refer mainly to the specific contexts analyzed. We selected testimonies that best illustrate the actors’ different perceptions on the determining conditions to perform EIR. Each testimony received an identifier to help read and interpret them. The countries and informants’ names were anonymized, keeping only those characteristics relevant for data interpretation. All testimonies presented here were translated into English from the Spanish transcripts.

We developed 36 codes for analysis and selected six main themes related to the categories of outer and inner settings of the CFIR (Appendix 2)^14^, also considered in the interview guide. We included other subthemes that emerged during data analysis and provided explanatory density.

### Ethical Considerations

The research protocol was approved by the Research Ethics Committee of the National Institute of Public Health of Mexico (CI-1454/02-2017). Each project principal investigator received an informed consent form by email, signing and returning it. An oral informed consent was required before each interview.

## RESULTS

Table 1 summarizes the main findings and recommendations of the seven projects, providing an overview of the health issues they addressed. The implementation problems identified and the recommendations suggested to solve them are consistent with the administrative level of the programs and policies analyzed; a relevant issue related to the feasibility of applying research results and recommendations.

The 15 iPIER initiative participants interviewed, 11 women and four men, were members of health public administration, health services or academic institutions. Interviewees included principal investigators, co-researchers and researchers participating in the research projects from various professional backgrounds: physicians, political sciences, psychology, industrial engineering, anthropology, bacteriology and sociology specialists (Table 2). Table 3 contains the description of the main themes and subthemes that emerged from the analysis, as well as all the related golden quotes.

**Table 3:** Major themes and subthemes with corresponding quotes.

Major themes		Subthemes		Golden quotes*	
1	Methods development and application	1.1	Before participating in the iPIER initiative, teams had no knowledge about EIR.	1.1.1	The first thing [we have gained] is being familiar with this Implementation Research theme, because none of us had any knowledge about it or knew this existed or what it is about. In a way, understanding it is a competence we have gained. PI, C4, 2°, M, CI, Medicine
		1.2	It is necessary to identify a research problem for which changes are feasible.	1.2.1	It is important to identify a problem that can be solved, which cannot be a structural problem, because, obviously, a structural problem cannot be solved with an implementation research (…). So, to identify the problem is also very important for this kind of research. (PC, C6, 1° & 2°, F, PI, Medicine)
		1.3	The use of qualitative methodology is needed to reach deep understanding.	1.3.1	(…) Qualitative research strengthens research (…) In P3, health issues are presented, at least in the media, in quantitative terms, which somehow makes data presentation a bit dark (…) Those complexities of the health world that are often not only explained with the analysis framework, but also using context and qualitative elements. (PG, C4, 2°, M, CI, Anthropology)
		1.4	It is necessary to consult implementers and health staff in the health policies, programs and services.	1.4.1	These people (implementers and health staff), as it is quite often, are for the first time consulted in an interview (…) it is not only that someone asks them, but that they can participate and express their point of view… I mean, I find the methodology interesting (…) And if it is not done with the actors of implementation, it is quite difficult to think that you can do something… (PB, C7, 2°, F, CI, Political Sciences)
		1.5	It is also important to consult the users of programs and services to inform about social and cultural factors affecting the implementation.	1.5.1	Pictures and videos of the nutritional sparks [Chispitas nutricionales] used to say: “Give it in a banana to the child,” but unfortunately in El Alto people don't eat bananas, mothers don't want to give bananas to their children because they believe this will cause caries in their teeth, so they don't give them bananas. Now they ask themselves, “With what should I give (the nutritional sparks) to them?” One mother told us: “I didn't give them to my child because the doctor said I should give them with banana, so I didn't give the sparks to him”. For me, these things are important. (PC, C6, 2°, F, PI, Medicine)
2	Timeline and human resources availability	2.1	EIR sometimes involves outsourcing researchers due to the implementers” workload.	2.1.1	It is very difficult to find a research team in a ministry. Usually, you find them in academia or, if you do find researchers, they are very busy working in administration activities, so it is not easy to find researchers doing qualitative research (…) basically, implementation processes need a lot of qualitative research, because of in depth interviews, focal groups or the methodology that is necessary to discover barriers and the such… Therefore, you can outsource that. (PA, C7, 1°, F, PI, Medicine)
		2.2	It is also necessary to generate motivation in the health staff collaborating in the research project.	2.2.1	To do the interviews, one must have time and needs to work after his or her working Schedule and sometimes the personnel is exhausted… and have many patients… (…) they have to fill in a bunch of papers for the health insurance, besides the clinical file. (…) So, we have to find the time to do the research. You need to be very motivated. You need a lot of motivation. (PC, C6, 1°, F, PI, Medicine)
		2.3	A strategic element to develop EIR is the profile of who performs it as well as the implementers’ involvement.	2.3.1	The decision maker's profile is essential, if (you choose) the basic personnel profile, one that has never made any research, the person will not be interested… this is the first thing… (…) A decision maker or an administrator who is not interested by academia [will complicate the research]. (PI, C3, 2°, F, CI, Medicine)
				2.3.2	The participation of the [regional decision maker] was very important, this gave us access to the health services (…) If the person in charge participates and facilitates things, this is very important, because [health personnel] usually don't like to give information. This is why we consider their participation as a priority, because, besides that, the results of this research can be applied. (PK, C2, 1° & 2°, F, CI, Medicine)
3	Financial and budgetary conditions	3.1	The funding allocated to EIR constitutes a basic condition for developing it.	3.1.1	To say the truth, even though budgets include research, it is very difficult to have access to these resources… (PO, C1, 2°, F, CI, Medicine)
				3.1.2	I think that this kind of implementation research is very feasible, because I think that the reason why not more of it is done is the lack of financial resources, but the authorities have shown good disposition and interest in it. (PN, C1, 2°, F, CO, Sociology)
				3.1.3	We currently have the financial support of PAHO, so, to do research you need a whole team, so we only have a very small amount to do research, which is not enough to hire this kind of experts, because you need experts… In some way, this would be one of our limitations. (PI, C3, 2°, F, CI, Medicine)
4	Institutional dynamics and organization	4.1	Some institutions have regulatory limitations to conduct research.	4.1.1	The Health Ministry does no research, the ministry finances research, this is an important point. XYZ is essentially a research institution; an important proportion of the research that the Ministry is interested in, is contracted with us. The ministry asks XYZ to do it. However, the Ministry, itself, does no research; it contracts partners to do it. (PE, C5, 2°, F, CI, Psychology)
		4.2	The instability of decision makers brings uncertainty, due to the lack of institutionalization of health policies and programs.	4.2.1	Unfortunately, in our country political will is too linked to the person holding a post. So, those who are currently responsible at the Public Health Ministry do have the political will related to the execution of this kind of logic in decision making. We cannot guarantee that if the people who are responsible for the process (change) this will continue to have the same acceptability. We, as a country, have certain limitations in our institutions that is hard to admit. (PM, C1, 1°, F, CI, Medicine)
				4.2.2	In (C4) we are very radical, there is no continuity in public policies… But, as I told you, if the political coalition that I think will win does win, they are going to put a lot of attention to this [medical drugs] policy according to their perspective and interests. (PG, C4, 2°, M, CI, Anthropology)
				4.2.3	The discontinuity of public officials who were acquainted with a line of action can have consequences. (PB, C7, 2°, F, CI, Political Sciences)
		4.3	Decision makers fear highlighting what doesn't work in their health programs or policies as well as the possibility of generating critical evidence.	4.3.1	National politics (…) are very important regarding this, when we have a very limiting government, we can have problems (…) There have been times in the country when programs were personalized, the person in charge was the owner, the god, and nobody could investigate him and say that things were not working, but this is not the case now, I think we are well. (PK, C2, 1°, F, CI, Medicine)
				4.3.2	[There is the need] to undo the idea that behind this they are a sanction, because if there is a sanction it must go through the right channels, it has nothing to do with any health research. (PB, C7, 2°, F, CI, Political Sciences)
				4.3.3	We also work with a very critical perspective of the freedom that a studies or research center gives us… It is different from a government institution… (PG, C4, 2°, M, CI, Anthropology)
5	Political environment	5.1	Health issues are prioritized according to political agendas and how they are addressed depends on the particular ideology of the ruling political group.	5.1.1	It does depend on political will (…) Nevertheless, now it is different. As I was telling you, the ministry belongs to the party holding power, but the municipality and the regional government are in the opposition… but are eager about it, because this an issue of interest, it is an issue related to the interest in the health of the population. (PC, C6, 1°, F, PI, Medicine)
				5.1.2	I think that first, if one puts in the agenda that the imprisoned population has the right to access healthcare, I think this is new vision, yes? (…) What one finds here is the result of years of policies… of not putting in the agenda, maybe, not health, but the penitentiary services of the province of XYZ, and this leads to a degradation in all senses (…) In my opinion, this has to do with a previous value consideration in relation to public policy that has been there for years (…) I believe this is a policies prioritization and to put the necessary funding and to work with a true sense of rights. (PB, C7, 2°, F, CI, Political Sciences)
				5.1.3	I believe that today, with the vision of the new coordinator, processes are being dismantled because she has a political mandate (…) If one of the leading lines of iPIER is the participation of health leaders, of the heads of health services, decision makers, I think that now the execution of this methodology (EIR) … I'm being sincere, may be difficult… (PD, C5, 1° & 2°, M, PI, Psychology)
6	Perception on the use of research results	6.1	Use of EIR results.	6.1.1	In the municipalities they do very little on health issues, they do very little while they could be taking a more important role in prevention and search for a solution (…) This is going to be a good tool at the national level that will help the local and regional authorities to act. (PK, C2, 2°, F, CI, Medicine)
				6.1.2	I believe that [with our research] the process [of the elaboration of a drug bioequivalence policy] will become visible since it worked, as little as it worked, but it did work… and I think that we will also generate inputs for more critical references for these processes so they can follow-up and monitor future policies in the domain that we have evaluated. (PG, C4, 2°, M, CI, Anthropology)
				6.1.3	It is very possible that the results be applied, the health system is strengthening its steering system, so this kind of research is timely and pertinent… in fact, the Health Ministry is currently in the process of clinical practice guides and protocols development. (PO, C1, 2°, F, CI, Medicine)
		6.2	Different strategies to apply EIR results.	6.2.1	We are still working in the second phase of the project, which consists of the establishment of a deliberative dialogue among social actors that really are responsible of the implementation of the TB prevention, treatment and control policy in the prisons of (our)the province… (PB, C7, 2°, F, CI, Political Sciences)
				6.2.2	I think that the results will help, but we have to present them in different decision-making levels, for instance, at the different health centers, this is super important (…) and to the regional authorities of the programs. (PC, C6, 2°, F, PI, Medicine)
				6.2.3	I have an advantage, because I will directly apply the findings that we have and the recommendations in my institution, this is the advantage, because I am the decisions maker, so I can already implement them. To implement it in the other [institutions of the city of XYZ], well I think it is necessary to sensitize the secretary of health in order to scale-up our strategy in other centers. (PI, C3, 2°, F, CI, Medicine)
		6.3	It is necessary to involve actors from different social sectors to generate changes in health programs and policies.	6.3.1	I believe that there is a lot of space to generate a link with certain sector of the [national] private sector in which certain general criteria can be deepened to face the most important economic power, which is represented by the international pharmaceutical industry that functioned as important social actors in the development of this policy (…). There are people in technical instances, people in the civil society, the medical and the chemical and pharmaceutical academies who are most critical about these processes. They are going to have close information of our analysis that will help to follow-up future processes. (PG, C4, 2°, M, CI, Anthropology)

* The testimony identifier was built as follows: PA = participant A, F/M = Female, Male, C1 = Country 1, PI/CI = Principal Investigator, Co-Investigator, Bachelor education.

XYZ: the names of the institutions and cities have been omitted in order to assure anonymity.

### 1. Methods development and application

In general, all participating teams lacked clarity regarding health systems research and, to a greater extent, implementation research at the beginning of research activities. Although some teams included experienced researchers or had them as partners for this project, such professionals usually had an epidemiological research orientation, having difficulty in understanding and adopting a health systems and implementation research perspective (quote 1.1.1).

Participants recognized that both during the research design phase and its results use, one must identify an implementation problem that can be solved with the available resources. More comprehensive problems related to structural issues are usually beyond the institutional influence capabilities of those conducting this type of research (quote 1.2.1).

Different informants mentioned that answering the research questions usually requires using qualitative research methods to reach the depth that will allow providing the correct answers and achieving the objectives (quote 1.3.1). And this is because EIR quite often requires direct consultation with program implementers to consider their experience (quote 1.4.1).

Similarly, several participants mentioned that it is equally important to gather the point of view of health service users to discover social and cultural factors that can affect the implementation of programs and policies (quote 1.5.1).

### 2. Timeline and human resources availability

Despite one of the main conditions of the EIR being that it must be led by implementers themselves, due to the heavy workload that implementation imposes on decision makers and frontline staff, participants recognized the need to outsource professional researchers to carry out some research activities (quote 2.1.1).

Likewise, considering these activities would be added to their regular tasks, interviewees recognized the need to generate a key motivation for the research and its results among the personnel participating in EIR projects (quote 2.2.1).

Several participants mentioned that the research team profile is a relevant issue, since its members need to know not only how the program or policy is implemented, but also how to do research (quote 2.3.1). In this sense, the inclusion of high-level decision makers in the research team appeared as strategic to facilitate fieldwork (quote 2.3.2).

### 3. Financial and budgetary conditions

Although iPIER teams received financial support to conduct their research projects, participants noted that the availability of financial resources is essential to conduct EIR (quote 3.1.1 & 3.1.2). The lack of resources allocated or earmarked for research, especially EIR, limits decision-makers’ and implementers’ capabilities to perform it, mainly because of the implicit workload in their roles and the lack of resources in their institutions (quote 3.1.3).

### 4. Institutional dynamics and organization

Participants recognize that, in certain circumstances, there are regulatory limitations to conducting research within government offices linked to program implementation. In such cases, the project outsourced research activities to academic institutions (quote 4.1.1).

According to the interviewees, the instability of some high-level decision makers’ positions is a contributing factor to the lack of institutionalization of certain health policies and programs and also affects EIR (quote 4.2.1, 4.2.2 & 4.2.3).

One aspect of the inner setting related to CFIR's implementation climate category is the belief that EIR could arouse suspicion among decision makers about their responsibility in implementing the program, as EIR could produce critical evidence on what is not working and, eventually, produce negative consequences for them. Ultimately, these situations may hinder the programs and EIR (quote 4.3.1, 4.3.2 & 4.3.3).

### 5. Political environment

Despite the relevance that EIR has at the national level when backed by international technical and financial support, according to some interviewees, the inclusion of certain health issues on the political agenda is a key aspect for improving policies and programs that can determine the possibilities of conducting EIR. In a way, health issues are prioritized according to their visibility to decision makers (quote 5.1.1).

But how one addresses a health issue (even the decision to address it) depends on the political perspective of the group in power (quote 5.1.2).

Sometimes research has to be reallocated due to the arrival of high-level decision makers with particular views on what health issues should be addressed and how (quote 5.1.3). These situations directly impact the possibility of conducting EIR and also determine being able to introduce changes in the programs based on research results.

### 6. Perception on the use of research results

Participants noted that EIR results can be used in different ways:

- As evidence to generate changes in the actions of program implementers (quote 6.1.1).
- As a contribution to different sectors of society lobbying for better public policies and generation of policy options (quote 6.1.2).
- As a useful tool to develop or improve healthcare guides and protocols (quote 6.1.3).

In this sense, interviewees mentioned different strategies that allow using EIR results to promote changes in the policies and programs studied, for example:

- Conduct deliberative dialogues with implementers (quote 6.2.1).
- Disseminate results at different decision-making levels (quote 6.2.2).
- Raise awareness among high-level decision makers on the relevance of the results (quote 6.2.3).

Finally, to create changes in health policies and programs based on EIR results, participants remarked the need to involve different social actors (quote 6.3.1).

## DISCUSSION

Our study points to different factors that can either facilitate or hinder EIR performance and the eventual use of its results to improve health policies, programs and services, mainly in the context of Latin America and the Caribbean. Focusing on the results of the 2016 IPIER initiative, this study first contrasts its findings with the published materials on the 2014 initiative, searching for new lessons. In general, one of the main barriers for conducting EIR is the lack of certain research capabilities among implementers. According to the EIR approach, these actors should lead the research efforts, emphasizing their prominence and collaboration with the professional researchers supporting them. This finding is consistent with the published literature^12^ and directly affects the development of research projects.

While the EIR approach contributes to improving decision-makers’ research capabilities, the goal is not to train them as researchers, but to improve their understanding of the usefulness of research evidence in improving health system performance^12^. Realizing the value of the EIR helps generate a sense of ownership of its results and implementers can identify them as a product of their own efforts^6^^,^^12^^,^^18^. This may even reduce the need for knowledge translation and promote immediate improvement of health policies and programs^12^^,^^19^^,^^20^.

Different participants agreed on the great advantage that having a high-level decision makers in the research team brings, echoing previous evidence in this regard^12^^,^^19^^,^^21^^,^^22^. Their administration skills facilitates executing EIR as well as the immediate or later use of results^12^. Although they usually represent a strategic force, under certain circumstances their participation can also become an obstacle. Among the most relevant are the great mobility of their posts (also causing the lack of program continuity), their heavy workload, and, eventually, their limited research capacity and experience^12^. Sometimes, these limitations were resolved by including professional researchers as close collaborators of the teams and, mainly, of the decision-makers.

When regulations prevented institutions from conducting research, direct relations with academic institutions and the outsourcing of professional researchers allowed to conduct the EIR projects. But with such research experts always working under the implementers’ leadership.

Although the projects in this study received financial support, interviewees repeatedly remarked the lack of resources to do research as a major limitation for the development and continuity of EIR in LAC^5^.

Participants highlighted political events as important phenomena that affect the health system and, consequently, the conditions in which EIR is developed. The 2014 iPIER participants also faced similar situations^23^^,^^24^. Such events are also related to the place health issues occupy in the political agendas and how they are prioritized in economic terms and in the media^25^. Similarly, the political views of some groups can influence not only how health issues are addressed, but even if they are addressed at all. The particular interests of social actors with economic power can also limit the possibilities to conduct EIR and use its results.

As such, the willingness to change and different political views and positions at the highest government levels or “political will,” as some informants describe it, can hamper implementers’ efforts to improve health programs. But as we identified in several testimonies, possibilities for using EIR results are related to what Reich^26^ describes as political feasibility. Social actors with enough power and capacity (civil society organizations, health professional associations, organized health services users, academic institutions or private entrepreneurs) can outweigh the opposition to address certain issues. Thus, most participating teams considered the advantages of including such actors to support the use of EIR results, as was successfully done by the 2014 IPIER teams^19^^,^^27^^,^^28^.

On other occasions, decision makers and health professionals are unwilling to collect information that may highlight deficiencies in the programs they operate^12^^,^^20^^,^^29^. This is certainly one of the many reasons why professional research is still very important and needs to be fostered within the academic domains where it is normally developed. External and independent research is the basis for objective assessments that cannot be addressed from the perspective of decision makers who have to provide quick and efficient answers to pressing issues.

Our study shows that the resources and support provided by the iPIER initiative were important facilitators of the EIR process. The initiative funded a neglected area of health research (implementation research) for which the participation of health staff is a crucial element^9^. And the technical advice and guidance it offered was based on an iterative and ongoing approach to capacity building, which helped decision makers and their teams develop a broader health system perspective, often absent in their backgrounds.

Another relevant issue is that while EIR aims to identify contextualized solutions to local implementation problems, it also allows to identify systemic health system dysfunctions that can be addressed by interventions at this higher and broader level. This points to a clear tension over smaller and easier to apply improvements in implementation (quick wins) versus longer-term changes in health system performance that are usually beyond the scope and responsibility of program managers.

Even if EIR aims to find solutions for implementation problems from the implementers’ point of view, the users’ perspective on what works and what can be improved is a recurring theme raised by the interviewees, as in the 2014 IPIER initiative^24^^,^^25^^,^^27^^,^^28^^,^^30^.

Strengthening the decision makers’ and implementers’ research capabilities increases the value and sense of ownership of EIR results among them, which can ensure greater and more direct use of scientific evidence to improve implementation and can shift the need for knowledge translation. EIR can be a good option to increase the relevance and impact of research. As it gains recognition, support and funding, more decision-makers will be willing to participate in this type of collaboration and apply research results to improve health system's performance.

Since no sufficient time has passed since the IPIER experience, the actual changes that could have been promoted by using the results of the implementation research conducted by the participating teams need further research, as is also true for the deepening of capacity building and policy collaboration to advance EIR.

In short, the timely application of certain strategies allows to overcome important barriers, make the most of facilitating factors and promote the use of results. Other challenges are beyond the influence of researchers, health professionals and even decision makers. But promoting and enabling the involvement of several social actors in research can be a powerful strategy in the hands of the health system itself.
